# The Frequency of Genetic Mutations Associated With Behavioral Variant Frontotemporal Dementia in Chinese Han Patients

**DOI:** 10.3389/fnagi.2021.699836

**Published:** 2021-07-08

**Authors:** Li Liu, Bo Cui, Min Chu, Yue Cui, Donglai Jing, Dan Li, Kexin Xie, Yu Kong, Tianxinyu Xia, Chaodong Wang, Liyong Wu

**Affiliations:** ^1^Department of Neurology, Xuanwu Hospital, Capital Medical University, Beijing, China; ^2^Department of Neurology, Shenyang Fifth People Hospital, Shenyang, China; ^3^Department of Neurology, Rongcheng People’s Hospital, Hebei, China; ^4^National Clinical Research Center for Geriatric Diseases, Beijing, China

**Keywords:** Chinese Han ethnicity, behavioral variant frontotemporal dementia, genetics, MAPT, GRN, C9orf72

## Abstract

**Background:**

Behavioral variant frontotemporal dementia (bvFTD) is a clinically heterogeneous syndrome with high heredity. However, the frequencies of mutations associated with bvFTD have yet to be determined. The aim of the current study was to investigate the frequency of Chinese Han patients harboring genetic bvFTD variants.

**Methods:**

A total of 49 bvFTD patients selected from our frontotemporal lobar degeneration database, including 14 familial cases belonging to eight families and 35 sporadic cases were consecutively recruited from July 2014 to December 2019 at Xuanwu Hospital (Beijing, China). Whole-exome sequencing (WES) was performed and repeat-primed PCR was used to test samples for the C9orf72 hexanucleotide repeat expansion mutation. The frequency of genetic variants and the pathogenicity of the novel variants were analyzed.

**Results:**

Ten pathogenic or likely pathogenic variants were identified in 17 bvFTD patients, including C9orf72 repeat expansions, six previously reported mutations and three novel mutations (MAPT p. R5C, p. D54N, GRN p. P451L). Genetic mutations accounted for 27.9% (12/43) of total cases, 87.5% (7/8) of patients with familial bvFTD, and 14.3% (5/35) with sporadic bvFTD. Pathogenic variants mostly occurred in MAPT gene (20.9%, 9/43), followed by C9orf72 repeat expansions (2.3%, 1/43), GRN gene (2.3%, 1/43) and FUS gene (2.3%, 1/43).

**Conclusion:**

There was a high prevalence of genetic variants in Chinese bvFTD patients, highlighting the necessity of genetic testing for bvFTD.

## Introduction

Frontotemporal lobar degeneration (FTLD) is a heterogeneous clinical syndrome with high heredity, which mainly includes behavioral variant frontotemporal dementia (bvFTD), non-fluent variant primary progressive aphasia (nfvPPA), and semantic variant primary progressive aphasia (svPPA) (Onyike and Diehl-Schmid, 2013; Ferrari et al., 2019; Seeley, 2019; Sirkis et al., 2019). Atypical forms of FTLD present an overlap with Parkinsonian disorders (PD), corticobasal syndrome (CBS) and progressive supranuclear palsy (PSP), and amyotrophic lateral sclerosis (FTD-ALS) (Ferrari et al., 2019). BvFTD, which presents as progressive abnormal changes in personality and social-emotional behavior, is the most common subtype of FTLD, and accounts for approximately 60% of all FTLD patients (Onyike and Diehl-Schmid, 2013; Seeley, 2019). Compared with the other two language variants, bvFTD has a stronger genetic component, almost 50% of patients with bvFTD have a positive family history compared to only 12% of patients with PPA (Greaves and Rohrer, 2019; Ramos et al., 2019a; Pytel et al., 2021). The genetics of FTLD have been studied widely, but the genetics of bvFTD remain elusive.

Known disease-causing genetic variants currently account for 10–40% of FTLD (Onyike and Diehl-Schmid, 2013; Sirkis et al., 2019). Mutations in hexanucleotide expansion repeats of C9orf72, microtubule-associated protein tau (MAPT), and granulin (GRN) have been definitively proven to be the most common types of pathogenic variants of FTLD, accounting for 6–30%, 3–14%, and 1–16%, respectively, in North American and European cohorts (Rohrer et al., 2009; Olszewska et al., 2016; Sirkis et al., 2019). In previous studies investigating FTLD genetics in Chinese populations the prevalence of pathogenic variants was comparatively lower (4.9–7.7%) (Tang et al., 2016; Che et al., 2017), but whether this discrepancy derives from geography and ethnicity factors remains to be clarified. Although bvFTD was included and analyzed in those aforementioned studies as the most important phenotype with the highest genetic mutation frequency, its genetic pathogenicity has never been focused on as an independent disease entity. In order to enhance understanding of bvFTD, specific genetic research investigating bvFTD is urgently needed.

In the current study whole-exome sequencing was performed, and the frequencies of pathogenic variants were analyzed in 49 Chinese Han bvFTD patients to investigate genetic features associated with bvFTD in this population.

## Materials and Methods

### Ethics Statement

The study was approved by the Ethics Committees of the Xuanwu Hospital of Capital Medical University, China, and was conducted in accordance with the principles stated in the Declaration of Helsinki. Written informed consent was obtained from each patient or their guardian.

### Participants

An FTLD database was established at the Department of Neurology of Xuanwu Hospital, China, which included 77 patients with FTLD who were consecutively recruited between July 1, 2014 and December 31, 2019. All patients underwent detailed clinical interviews, physical examinations, neuropsychological assessments, cerebral 18F-fluorodeoxyglucose positron emission tomography/magnetic resonance imaging examinations (18F-FDG PET/MRI), and genetic testing within 1 month of recruitment. The diagnosis was performed according to the consensus criteria for probable bvFTD published in 2011, which requires three out of six clinically discriminating features (disinhibition, apathy/inertia, loss of sympathy/empathy, perseverative/compulsive behaviors, hyperorality, and dysexecutive neuropsychological profile), functional disability, and characteristic neuroimaging (Rascovsky et al., 2011). Each patient was followed up for at least 1 year. In total 49 patients enrolled in the final analysis met the diagnostic criteria for probable bvFTD. The family history of each patient was analyzed by assigning a modified Goldman score between 1 and 4, as described previously (Goldman et al., 2005).

### Genetic Screening

We extracted genomic DNA from all patients enrolled in the study from fresh peripheral blood leukocytes and used an Agilent SureSelect Human All Exon V6 Kit (Agilent Technologies, Santa Clara, CA, United States) to generate a sequencing library for whole exome sequencing (WES). The prepared libraries were sequenced using the HiSeq-2000 platform (Illumina, San Diego, CA, United States). The sequenced reads were aligned to the human genome (GRCh37/hg19). Reads were then aligned to the targeted regions and collated for single nucleotide polymorphism (SNP) calling and subsequent analysis using Burrows-Wheeler Aligner software. All potential variants were verified via Sanger sequencing, which was performed on an ABI3730xl Genetic Analyzer (Applied Biosystems). Repeat primed PCR was performed as previously described to obtain a qualitative estimation of the presence of C9orf72-expanded repeats (Tang et al., 2016). Next, ANNOVAR software and Realigner Target Creator in Genome Analysis Toolkit were used to annotate the variants (McKenna et al., 2010; Wang et al., 2010).

We explored variants from genes associated with ‘‘dementia’’ according to several databases: Human Gene Mutation Database (HGMD^1^), Online Mendelian Inheritance in Man (OMIM^2^), Clinvar^3^, and GeneCards^4^. All shortlisted genes that were associated with dementia were verified by UniProt^5^, a web resource which curates comprehensive, high-quality, annotated information of a gene with its corresponding protein functions. The selected genes were further confirmed by MalaCards^6^, an integrated database of public literatures relating to human disease and disorders. Our final analysis included 42 genes that were associated with FTD and other neurodegenerative diseases. Supplementary Table 1 provides further details of the genes selected for analysis.

### Variant Assessment

Variants were filtered for missense, nonsense, splice site, frameshift, non-frameshift. The splicing site was defined as ± 1 or 2 bp of the splicing donor or acceptor sequence. To prioritize variants, we used a stringent minor allele frequency filter (<1%) in several public databases: the single-nucleotide polymorphism database^7^, the 1000 Genomes Project^8^, the ExAC database^9^, and the genome aggregation database^10^. *In silico* prediction of the functional effects of missense mutations was conducted using Polymorphism Phenotyping v.2 (PolyPhen2) (Adzhubei et al., 2010), Sorting Intolerant From Tolerant (SIFT) (Choi, 2012), MutationTaster (Schwarz et al., 2014), and the likelihood ratio test (LRT) (Chun and Fay, 2009). Protein sequence alignment was performed with UniProt^11^ to determine whether sequences were evolutionarily conserved across different species including *Mus musculus* (mouse), *Rattus norvegicus* (rat), *Equus caballus* (horse), *Felis catus* (cat), *Bos taurus* (bovine), and *Macaca mulatta* (rhesus macaque). All analyses were performed on the Seqmax platform^12^ and Pubvar platform^13^ platforms. Significant findings were comprehensively assessed by considering minor allele frequency (MAF), predicted pathogenicity, disease association, and family history.

### Statistical Analysis

The frequency of genetic mutations was calculated as the number of patients with mutations relative to the total number of samples with and without mutations. The frequency of each mutation was equal to the number of patients with the mutation of interest relative to the total sample size with and without a given mutation. Statistical analysis was performed using GraphPad Prism v.8.0. Categorical variables were compared by the Chi-squared test. Data are represented as means ± the standard deviation. Two-tailed *p* values < 0.05 were considered statistically significant.

## Results

### Demographic Features of the Subjects

Of the 49 bvFTD patients identified in our final dataset (25 males; 51.0%), 14 patients from eight families were identified as having familial bvFTD (f-bvFTD), while 35 were considered to have sporadic bvFTD (s-bvFTD). The demographic features of these patients are shown in Supplementary Table 2. Family history was positive in 14 out of 49 cases (28.6%), with modified Goldman scores of 1.0–3.5 and a mean modified Goldman score of 1.9. There were no significant differences between the f-bvFTD and the s-bvFTD patients in terms of gender, age at onset, years of education, onset-diagnosis interval, MMSE, MoCA, CDR, NPI-Q, MBI-C, or FBI scores between f-bvFTD and s-bvFTD patients.

### Genetic Screening

Whole-exome sequencing was conducted in all 49 bvFTD patients in the study. The mean sequencing depth for target regions was 125.48×. On average, per sequencing individual, 99.87% of targeted bases were covered by at least 1 × coverage and 99.50% of the targeted bases had at least 10 × coverage. Many rare variants were identified in bvFTD patients. However, we focused particularly on variants in genes with known functions that were potentially associated with FTD and other neurodegenerative diseases, such as PD, ALS, and AD. After filtering (as described above) and confirmed via Sanger sequencing, nine possible pathogenic variants were identified in 15 patients, including six known pathogenic variants (MAPT p. P301L, p. N279K, p. V337M, p. N296N, p.P513A; FUS p. G231del), and 3 novel variants (MAPT p. R5C, p. D54N; GRN p. P451L). None of these pathogenic variants had been reported previously in the public databases described above. C9orf72 repeat expansions (>52 repeats) were observed in the two additional patients. All of the detected pathogenic variants and clinical features are shown in Supplementary Table 3.

### Variant Assessment

**FIGURE 1:**
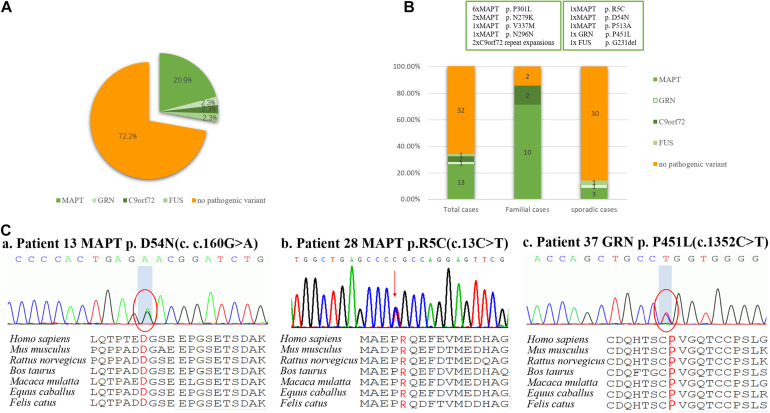
Frequency of mutations in a consecutive series of 49 subjects with behavioral variant frontotemporal dementia. **(A)** 27.9% of subjects carried mutations, including those of MAPT, GRN, FUS, and C9orf72 repeat expansion, but surprisingly not CHCHD10. **(B)** Mutations were found in 87.5% of familial subjects and 14.3% of sporadic subjects. **(C)** Sanger sequencing revealed two novel missense mutations of MAPT (p. R5C and p. D54N), and one novel missense mutation of GRN (p. P451L). These missense mutations were present at a highly conserved position, as indicated by a comparison of the corresponding sequences of seven vertebrate species.

Details relating to novel variants, along with pathogenic prediction are summarized in Table 1 and Figure 1C. Three novel variants were absent in the general population. These missense variants affected an amino acid that had been highly conserved during evolution, and were classified as “possibly damaging,” “damaging,” or “disease causing” by the three prediction programs PolyPhen2, SIFT, and MutationTaster, respectively. In addition, the MAPT R5C variant was a missense mutation in the same amino acid location as other pathogenic variant (MAPT R5H/R5L) that had been reported previously (Lin et al., 2017).

**TABLE 1:** Pathogenicity prediction analyses of three novel missense variants identified in the study.

**Mutation**	**Family history**	**Genomic location (hg19)**	**Location in the gene**	**Reference sequence**	**Reference SNP (rs) ID**	**Amino acid change**	**MAF**	**Database**			**Pathogenicity**				
								**Genome Aggregation Database**	**1000 Genomes Project**	**Exome Aggregation Consortium**	**Polyphen2**	**SIFT**	**Mutation Taster**	**LRT**	**CADD score**
MAPT c.160G > A	–	17:44049251	Exon 3	NM_016 835.4	–	p. D54N	absent	NR	NR	NR	Probably damaging	Damaging	Disease causing	Deleterious	26.6
MAPT c.13C > T	–	17:44039716	Exon 1	NM_0168 35.4	rs7661 66210	p. R5C	absent	NR	NR	NR	Probably damaging	Damaging	Disease causing	Neutral	34.0
GRN c.1352C > T	–	17:42429555	Exon 11	NM_002 087.2	rs7524 28000	p. P451L	absent	NR	NR	NR	Probably damaging	Damaging	Disease causing	Deleterious	34.0

*MAF, minor allele frequency; CADD, combined annotation-dependent depletion; LRT, likelihood ratio test; NR, not reported; Polyphen2, Polymorphism Phenotyping v.2; SIFT, Sorting Intolerant From Tolerant.*

### Frequency of Mutations

Of the 49 patients, 2 carried a pathogenic C9orf72 repeat expansion, 12 carried known gene mutations, 3 carried novel mutations, and 32 did not carry any mutations. Ten pathogenic or likely pathogenic variants were identified, accounting for 27.9% (12/43) of total cases, 87.5% (7/8) patients with f-bvFTD and 14.3% (5/35) with s-bvFTD (Figures 1A,B).

Mutations in MAPT were the most common genetic determinant, with a mutation frequency of 20.9% (9/43) in all bvFTD patients, 75% (6/8) in f-bvFTD patients, and 8.6% (3/35) in s-bvFTD patients. C9orf72 repeat expansion was detected in 2.3% (1/43) of bvFTD patients. In addition, GRN and FUS mutation was responsible for 1 bvFTD case (2.3%), respectively. Nevertheless, no mutations in CHCHD10, VCP, TARDBP, or TBK1 genes were detected.

Of the patients with MAPT mutations, six patients (46.2%) from three families carried P301L mutation, and two patients (15.4%) from one family had an N279K mutation. V337M, N296N, R5C, D54N, and P513A were also detected in five patients with MAPT mutations, respectively (7.7%).

### Clinical Features of Patients Carrying Mutations

Genetic, clinical, and imaging features of the seventeen clinical probable bvFTD patients with mutations identified in this study are summarized in Supplementary Table 3. There were statistically significant differences in terms of age at onset when comparing patients with mutations and those who did not have any of these mutations (57.76 ± 9.17 versus 64.81 ± 9.08; *t* = 2.56; *p* = 0.014). Most patients (12, 70.6%) exhibited personality changes and inappropriate behaviors at onset, and five (29.4%) patients presented with memory decline (Supplementary Table 3).

Of the 17 variant carriers, extrapyramidal signs occurred in 1 MAPT P301L variant, 2 MAPT N279K variants, and 2 C9orf72 repeat expansions, which were from three families, respectively. The first one belonged to the bvFTD family, in which 2 generations of 4 patients with bvFTD were found to be associated with a known mutation in MAPT p.P301L. All patients exhibited typical symptoms of bvFTD, except for one male patient who developed the disease at the age of 67 years, with Parkinsonism as the main manifestation, accompanied by mild cognitive decline. Two affected individuals suffering from bvFTD with parkinsonism were found in the pedigree with C9orf72 repeat expansion, in which more than 50 repeats were observed. The proband was a 66-year-old woman that presented with Parkinsonism as the initial symptom of onset and was misdiagnosed as Parkinson’s disease. Her younger brother developed behavioral changes and cognitive impairment with gradual onset at 62 years-of-age. One year later, the patient experienced progressive bradykinesia and rigidity.

## Discussion

To the best of our knowledge this is the first investigation of genetic features in well-characterized Chinese Han bvFTD patients. In this study potentially pathogenic variants were detected in 27.9% of the bvFTD patients, and the most frequently affected gene was MAPT. In total, we identified two novel MAPT mutations and one novel GRN mutation were identified. These findings indicate that genetic mutations are relatively common in Chinese bvFTD patients.

Two novel MAPT variants (p. R5C, p. D54N) and one novel GRN variant (p. P451L) were identified in the current study. All three missense mutations were absent from the gnomAD, 1000 Genomes Project, and Exome Aggregation Consortium databases. Amino acids in this region are highly conserved across several species, and the variants identified were predicted to be damaging by four *in silico* analysis tools. All of the patients harboring these novel variants exhibited the classic manifestations of bvFTD, including personality change, inappropriate behaviors, and significant hypometabolism in frontal and temporal lobes as determined by 18F-FDG-PET. Apart from these, the MAPT R5C variant was located in exon 1 of the MAPT gene, where the other two known mutations (MAPT R5H and R5L) have been found in Japanese and Taiwanese bvFTD patients (Lin et al., 2017). Collectively, three novel variants may be associated with bvFTD in the Chinese population, further expanding the known mutational spectrum of bvFTD.

The frequency of mutations in Chinese bvFTD patients was higher than expected. In the present bvFTD cohort genetic mutations accounted for 27.9% of bvFTD cases, and mutations were more likely to be detected in patients with a definite family history of bvFTD (87.5%). Mutations were also detected in sporadic cases (14.3%). In previous studies, bvFTD was usually included and analyzed as the most important phenotype of FTLD (summarized in Table 2), which did not specifically focus on bvFTD. Consequently, the prevalence of pathogenic variants in Chinese bvFTD patients has not been reported. In the only three existing studies based on Chinese frontotemporal dementia (FTD) cohorts, the prevalence of pathogenic variants was comparatively low (4.9–7.7%) (Shi et al., 2016; Tang et al., 2016; Che et al., 2017). A number of explanations may account for the discrepancy between these previously reported rates and the comparatively higher frequency of genetic mutations observed in the current study. The high proportion of f-bvFTD (28.6%) identified in the present study, compared to the 13.5% reported previously (Tang et al., 2016) was likely to have been contributory, because a higher frequency of mutations was detected in bvFTD patients with a family history. The discrepancy may also be the result of pure bvFTD phenotype screening criteria depending on FDG-PET in the present study, which was more heritable than the language syndromes nfvPPA and svPPA. There may also be differences between subjects from Northern and Southern China, as notably most of the aforementioned previous studies were based in South China (Tang et al., 2016; Che et al., 2017; Jiang et al., 2021). In any event, in view of the substantial proportion of mutations discovered in the present cohort, genetic screening should be considered in all bvFTD patients – even those without a family history of dementia – because underlying genetic causes of bvFTD cannot be excluded.

**TABLE 2:** Identified mutations in Chinese bvFTD patients.

**References**	**Gene**	**Nucleic acid change**	**Amino acid change**	**Area**	**Phenotype**			
					**Behavioral changes**	**Cognitive impairment**	**Parkinsonism**	**Motor syndrome**
Che et al., 2017	MAPT	c.14G > A	p.R5H	Shanghai	NA	NA	NA	NA
Tang et al., 2016		c.837T > G	p.N279K	Hunan/Beijing	+	+	+	−
He et al., 2018		c.902C > T	p.P301L	Henan/Tianjin	+	+	−	−
Xu et al., 2018		c.1165G > A	p.G389R	Shanghai	+	+	+	−
Che et al., 2017	GRN	c.750C > A	p.D250E	Shanghai	+	NA	NA	NA
Tang et al., 2016		c.898C > T	p.Q300X	Hunan	+	+	−	−
Jiao et al., 2014	C9orf72	GGGGCC hexanucleotide repeat expansion		Hunan	+	+	−	−
Jiao et al., 2016	CHCHD10	c.64C > T	p.H22Y	Hunan	+	+	−	−
Jiao et al., 2016; Che et al., 2017		c.67C > T	p.P23S	Hunan/Shanghai	+	+	−	−
Jiao et al., 2016		c.95C > A	p.A32D	Hunan	+	NA	−	−
Jiao et al., 2016		c.170T > A	p.V57E	Hunan	+	+	−	−
Che et al., 2017		c.266C > T	p.P89L	Hunan	NA	NA	NA	NA
Shi et al., 2016	VCP	c.379A > G	p.T127A	Tianjin	+	+	+	−
Yu et al., 2019	TBK1	c.1001T > C	p.I334T	Shanghai	−	+	−	−
Tsai et al., 2016		c.1330C > T	p.R444X	Taiwan	+	+	−	+

The frequency of MAPT variants in the present cohort was 20.7%, and they were more common in bvFTD patients with a positive family history, followed by C9orf72 repeat expansions (2.3%), disease-associated variants of GRN (2.3%) and FUS (2.3%). High variability in mutation prevalence across different geographical regions has been reported for C9orf72, MAPT, and GRN genes, and hexanucleotide repeat expansions in C9orf72 are the most common genetic cause of bvFTD in western populations (Seeley, 2019). The most common genetic determinant in the present bvFTD cohort was MAPT mutations, and C9orf72 repeat expansions were identified with a low frequency, which differ from reports derived from Europe and America (Greaves and Rohrer, 2019; Oijerstedt et al., 2019; Ramos et al., 2019b, 2020), but are similar to previous studies involving Chinese patients with FTD (Shi et al., 2016; Tang et al., 2016; Che et al., 2017; Jiang et al., 2021). Notably, the frequency of MAPT mutations was much higher than described in previous studies of Chinese FTD patients (2.8%) (Jiang et al., 2021). The most common MAPT mutation in the present study was P301L, which was not clear in a previous study in Chinese FTD patients. This may be a consequence of the high proportion of f-bvFTD patients in the current study since the P301L mutation was only detected in subjects with a family history of bvFTD.

Interestingly, Jiao et al. (2016) reported that rare variants in the CHCHD10 gene were detected at high frequencies in Chinese sporadic FTD patients, some of which were present in subjects with bvFTD. No mutations in CHCHD10 were identified in the current cohort, indicating that they are likely to be either not causative or a rare cause of bvFTD. The relatively small sample size, pure bvFTD phenotypes, and different geographical study region in the present study may also have contributed to this discrepancy. It is notable that these populations and geographical differences have to be interpreted with caution, because they may also be partly influenced by between-center and between-region differences in subject recruitment. Therefore, further screening of larger bvFTD cohorts needs to be undertaken in different geographical areas.

Though there were no significant differences in terms of age at onset between f-bvFTD and s-bvFTD patients, the frequency of mutations was significantly higher in a definite family history of bvFTD compared with sporadic cases (87.5% versus 14.3%). Moreover, age at onset was earlier in bvFTD patients with mutations than those without mutations. A younger age at onset and a strong positive family history were particularly strong predictors for the presence of a pathogenic mutation, which may be relevant in guiding the priority of genetic testing for this population in clinical practice.

Notably, prominent parkinsonism was found for the first time in Chinese bvFTD patients with the P301L MAPT and C9orf72 gene mutation, respectively. It is well documented that the P301L MAPT mutation and C9orf72 repeat expansion were are predominantly associated with FTD in the western population, often accompanied by Parkinsonism (Siuda et al., 2014). In the Chinese cohort, a variety of phenotypes with MAPT mutation were observed, which was associated with mutation location to some extent (Shi et al., 2016; Tang et al., 2016; Che et al., 2017; Mao et al., 2021). However, prominent Parkinsonism has not been reported previously for the P301L MAPT mutation (Shi et al., 2016; He et al., 2018). In the present cohort, one of four affected individuals in the pedigree with P301L MAPT mutation presented with Parkinsonism. This demonstrated the phenotypic variability associated with the P301L mutation in individuals with the same MAPT mutation, even within the same family. GGGGCC repeat expansions in the C9orf72 gene is rarely detected in Chinese FTD patients. A search of the existing literature showed that C9orf72 repeat expansion has only previously been identified in a patient with sporadic bvFTD and a family with FTD-ALS (Jiao et al., 2014). Interestingly, GGGGCC repeat expansions in the C9orf72 gene was found in a family with FTD and Parkinsonism in the present study, thus suggesting that parkinsonism in affected individuals may be attributable to dopaminergic dysfunction of the putamen resulting from C9orf72 repeat expansion. In contrast with Europe, parkinsonism is relatively rare in the FTD patients possessing the P301L MAPT mutation or the C9orf72 repeat expansion in China, thus suggesting that genetic heterogeneity is associated with different geographical regions and ethnicities. These results indicate that parkinsonism might be present in the pure bvFTD phenotype with P301L MAPT mutation or C9orf72 repeat expansion in China, thus suggesting that more attention should be paid to parkinsonism in patients with bvFTD in clinical practice.

The current study had some limitations. One was the relatively small sample size, and another was that gene recognition was based on clinical diagnosis of bvFTD patients rather than postmortem autopsy-based diagnosis, which affected the acquisition of conclusive information relating to the clinical manifestations and pathogenesis of mutations. We will address this issue via pathological evaluation and mutation function analysis in the future.

## Conclusion

In summary, in the present study potentially pathogenic variants were detected in approximately 28% of bvFTD patients, and MAPT variants were the most common causative genetic factors. Three novel bvFTD-related pathogenic variants were identified (MAPT p. R5H, p. D54N, and GRN p. P451L), broadening the known mutation diversity of bvFTD. The study provides evidence of a high prevalence of pathogenic variants in Chinese bvFTD patients, and highlights the necessity of genetic screening for bvFTD.

## Data Availability Statement

According to national legislation/guidelines, specifically the Administrative Regulations of the People’s Republic of China on Human Genetic Resources (http://www.gov.cn/zhengce/content/2019-06/10/content_5398829.htm, http://english.www.gov.cn/policies/latest_releases/2019/06/10/content _281476708945462.htm), no additional raw data is available at this time. Data of this project can be accessed after an approval application to the China National Genebank (CNGB, https://db.cngb.org/cnsa/). Please refer to https://db.cngb.org/, or email: CNGBdb@cngb.org for detailed application guidance. The accession code CNP0001883 should be included in the application.

## Ethics Statement

The studies involving human participants were reviewed and approved by the Ethics Committee of Xuanwu Hospital of Capital Medical University, China. The patients/participants provided their written informed consent to participate in this study.

## Author Contributions

LL and LW were responsible for study concept and design. YC, DJ, DL, KX, YK, and TX were responsible for clinical data collection. MC and KX were responsible for sequencing and quality control. YC, MC, and CW analyzed gene results. LL and BC were mainly responsible for writing the manuscript. CW and LW were responsible for revising the manuscript for important intellectual content. All authors read and approved the final manuscript.

## Conflict of Interest

The authors declare that the research was conducted in the absence of any commercial or financial relationships that could be construed as a potential conflict of interest.

## Footnotes

1 http://www.hgmd.cf.ac.uk/ac/search.php

2 https://www.omim.org/

3 https://www.ncbi.nlm.nih.gov/clinvar

4 https://www.genecards.org

5 http://www.uniprot.org/

6 http://www.malacards.org/

7 https://www.ncbi.nlm.nih.gov/snp/

8 https://www.ncbi.nlm.nih.gov/variation/tools/1000genomes/

9 https://exac.broad.institute.org/

10 http://gnomad.Broad.institute.org/

11 https://www.uniprot.org

12 https://www.seqmax.com/ accessed March 2020

13 https://www.pubvar.com accessed March 2020
