# Supplementary material for: The Frequency of Genetic Mutations Associated With Behavioral Variant Frontotemporal Dementia in Chinese Han Patients
Source: Front Aging Neurosci. 2021 Jul 8;13:699836. doi: 10.3389/fnagi.2021.699836 (PMC8297439; doi:10.3389/fnagi.2021.699836)
Supplement: Supplementary file 1 [file Data_Sheet_1.docx]

Supplementary Material

# Supplementary Table 1: The selective gene for analyses

| Classification | Gene list |
| --- | --- |
| Frontotemporal Dementia-associated gene | MAPT, GRN, TARDBP, FUS, VCP, CHMP2B, SQSTM1, CHCHD10, TBK1, UBQLN2, OPTN, CCNF, TIA1, hnRNPA1, hnRNPA2B1 |
| Other neurodegeneration-associated gene | PSEN1, PSEN2, APP, TREM2, DCTN1, SIGMAR1, hnRNPA2B1, GBA, NOTCH3, TRPM7, ABCC1, ABCA7, APBB2, ATP13A2, SPG21, DMT1, VPS2B, ALS17, EIF4G1, SCN8A, COQ2, TSC1, TSC2, HCFC1, ITPR3, PLA2G6, CYLD |

# Supplementary Table 2: Demographic and neuropsychiatric assessment data

|  | f-bvFTD (*n* = 14) | s-bvFTD (*n* = 35) | t/χ^2^ value | *p* value^*^ |
| --- | --- | --- | --- | --- |
| Age | 60.79 ± 3.14 | 61.91± 1.78 | 0.327 | 0.745 |
| Sex (male/female) | 6/8 | 19/16 | 0.723 | 0.522 |
| Years of education | 8.46 ± 1.74 | 10.37 ± 0.83 | 1.122 | 0.270 |
| Age at onset | 58.07 ± 2.92(40-75) | 59.54 ± 1.75 (25-78) | 0.443 | 0.660 |
| Onset-diagnosis interval | 3.16 ± 0.56 | 2.22 ± 0.27 | 1.634 | 0.110 |
| MMSE | 18 ± 2.63 | 17.91 ± 0.98 | 0.039 | 0.970 |
| MoCA | 14 ± 2.52 | 11.76 ± 0.94 | 1.039 | 0.307 |
| BNT | 8.50 ± 1.5 | 11.77 ± 1.22 | 0.788 | 0.439 |
| CDR | 1.63 ± 0.55 | 1.57 ± 0.18 | 0.121 | 0.905 |
| NPI-Q | 41.8 ± 14.34 | 24.29 ± 3.06 | 1.919 | 0.067 |
| MBI-C | 32 ± 18 | 25.67 ± 3.65 | 0.585 | 0.569 |
| FBI | 33 ± 8.50 | 31.31 ± 2.84 | 0.245 | 0.807 |

Data are presented as means ± the standard deviation.

*Two-sided p values for continuous variables refer to unpaired t-tests, two-sided p values for categorical variables refer to Pearson’s Chi-squared test.

BNT, Boston Naming Test; CDR, Clinical Dementia Rating; FBI, Frontal Behavioral Inventory; f-bvFTD, familial behavioral variant frontotemporal dementia; MBI-C, Mild Behavioral Impairment Checklist; MMSE, Mini-Mental State Examination; MoCA, Montreal Cognitive Assessment; NPI-Q, Neuropsychiatry Inventory Questionnaire; s-bvFTD, sporadic behavioral variant frontotemporal dementia

# Supplementary Table 3: Genetic, clinical, and imaging features of the seventeen clinical probable behavioral variant frontotemporal dementia patients with pathogenic variants identified in the study.

| Reference | Family number | Gene | Nucleotide change/ repeat expansions | Reference SNP (rs) ID | Amino acid change | MAF | Effect (HGMD) | AAO | FH | First clinical symptom(s) | MRI | 18F-FDG-PET  (hypometabolism) |
| --- | --- | --- | --- | --- | --- | --- | --- | --- | --- | --- | --- | --- |
| ^(1)^ | ^a^1-1 | MAPT | c.837T>G | rs63750756 | p. N279K^e^ | absent | DM | 40 | + | parkinsonism | normal | bilateral frontal, temporal, occipital cortex  R>L |
| ^(1)^ | ^a^1-2 | MAPT | c.837T>G | rs63750756 | p. N279K^e^ | absent | DM | 46 | + | parkinsonism/  behavior change | bilateral frontal and temporal atrophy | bilateral frontal, temporal, occipital cortex  L>R |
| ^(2)^ | ^b^2-1 | MAPT | c.902C>T | rs63751273 | p. P301L^e^ | absent | DM | 54 | + | behavior change | bilateral frontal and temporal atrophy | bilateral frontal, temporal cortex  R>L |
| ^(2)^ | ^b^2-2 | MAPT | c.902C>T | rs63751273 | p. P301L^e^ | absent | DM | 68 | + | behavior change | NA | NA |
| ^(2)^ | ^b^2-3 | MAPT | c.902C>T | rs63751273 | p. P301L^e^ | absent | DM | 53 | + | behavior change | NA | NA |
| ^(2)^ | ^b^2-4 | MAPT | c.902C>T | rs63751273 | p. P301L^e^ | absent | DM | 64 | + | parkinsonism | NA | NA |
| ^(3)^ | 5 | MAPT | c.1009G>A |  | p. V337M^e^ | absent | DM | 52 | + | behavior change | bilateral frontal and temporal atrophy | bilateral frontal, temporal cortex |
| ^(4)^ | 7 | MAPT | c.888T>C | rs63750912 | p. N296N^e^ | absent | DM | 45 | + | behavior change | bilateral frontal atrophy | bilateral frontal cortex |
| Present study | 13 | MAPT | c.160G>A | - | p. D54N^d^ | absent | DM | 67 | - | behavior change/memory decline | bilateral frontal and temporal atrophy, L>R | bilateral frontal, temporal cortex |
| ^(2)^ | 19 | MAPT | c.902C>T | rs63751273 | p. P301L^e^ | absent | DM | 52 | + | behavior change | NA | NA |
| ^(2)^ | 25 | MAPT | c.902C>T | rs63751273 | p. P301L^e^ | absent | DM | 64 | + | behavior change | bilateral frontal and temporal atrophy | NA |
| Present study | 28 | MAPT | c.13C>T | rs766166210 | p. R5C^d^ | absent | DM | 44 | - | behavior change/memory decline | left frontal atrophy | bilateral frontal, temporal, occipital cortex |
| ^(5)^ | ^c^29-1 | C9orf72 | GGGGCC repeats (>52) |  | NA | NA | DM | 65 | + | behavior change | bilateral frontal and temporal atrophy | bilateral frontal, temporal, occipital cortex |
|  | ^c^29-2 | C9orf72 | GGGGCC repeats (>52) |  | NA | NA | DM | 69 | + | parkinsonism | bilateral frontal and temporal atrophy | bilateral frontal, temporal, occipital cortex |
| ^(6)^ | 36 | FUS | c.684_686delCGG | rs760734999 | p. G231del^e^ | absent | DM | 62 | - | memory decline/  motor syndrome | bilateral frontal atrophy | bilateral frontal cortex |
| Present study | 37 | GRN | c.1352C>T | rs752428000 | p. P451L^d^ | absent | DM | 52 | - | behavior change/memory decline | bilateral frontal atrophy | bilateral frontal cortex |
| ^(7)^ | 40 | MAPT | c.1537C>G | rs267604921 | p. P513A^e^ | absent | DM | 60 | - | memory decline | bilateral frontal and temporal atrophy | bilateral frontal, temporal cortex |

^a^Family 1: Two patients with MAPT N279K variants were identified in this FTD-parkinsonism family.

^b^Family 2: Two generations of four patients with bvFTD were identified as being associated with a known mutation in MAPT p. P301L. One of them presented with Parkinsonism as the first clinical symptom.

^c^Family 29: Two patients with C9orf72 repeat expansions were identified in this FTD-parkinsonism family.

^d^Novel mutations identified in this study

^e^Known mutations

18F-FDG-PET, positron emission tomography of 18F-fluorodeoxyglucose; AAO, age at onset; FH, family history; L, left; MRI, magnetic resonance imaging; NA, not available; R, right

Reference

Wu L, Liu J, Feng X, Dong J, Qin W, Liu Y, et al. 11CCFT-PET in presymptomatic FTDP-17: a potential biomarker predicting onset. J Alzheimers Dis. 2018;61: 613–8.

He S, Chen S, Xia MR, Sun ZK, Huang Y, Zhang JW. The role of MAPT gene in Chinese dementia patients: a P301L pedigree study and brief literature review. Neuropsychiatr Dis Treat. 2018;14:1627–33.

Spina S, Schonhaut DR, Boeve BF, Seeley WW, Ossenkoppele R, O'Neil JP, et al. Frontotemporal dementia with the V337M *MAPT* mutation: Tau-PET and pathology correlations. Neurology. 2017;88:758-766.

Spillantini MG, Yoshida H, Rizzini C, Lantos PL, Khan N, Rossor MN, et al. A novel tau mutation (N296N) in familial dementia with swollen achromatic neurons and corticobasal inclusion bodies. Ann Neurol. 2000;48:939-43.

Jiao B, Tang B, Liu X, Yan X, Zhou L, Yang Y, et al. Identification of C9orf72 repeat expansions in patients with amyotrophic lateral sclerosis and frontotemporal dementia in mainland China. Neurobiol Aging. 2014;35: 936.e19–22.

Kwon MJ, Baek W, Ki CS, Kim HY, Koh SH, Kim JW, et al. Screening of the SOD1, FUS, TARDBP, ANG, and OPTN mutations in Korean patients with familial and sporadic ALS. Neurobiol Aging. 2012;33:1017.e17-23.

Tang M, Gu X, Wei J, Jiao B, Zhou L, Zhou Y, et al. Analyses MAPT, GRN, and C9orf72 mutations in Chinese patients with frontotemporal dementia. Neurobiol Aging. 2016;46:235.e11–15.
